# Applying Genome-Resolved Metagenomics to Deconvolute the Halophilic Microbiome

**DOI:** 10.3390/genes10030220

**Published:** 2019-03-14

**Authors:** Gherman Uritskiy, Jocelyne DiRuggiero

**Affiliations:** Department of Biology, Johns Hopkins University, Baltimore, MD 21218, USA; guritsk1@jhu.edu

**Keywords:** extremophiles, halophilic microorganisms, hypersaline habitats, metagenomics, microbiome, shotgun sequencing, genome assembly and binning, functional annotation

## Abstract

In the past decades, the study of microbial life through shotgun metagenomic sequencing has rapidly expanded our understanding of environmental, synthetic, and clinical microbial communities. Here, we review how shotgun metagenomics has affected the field of halophilic microbial ecology, including functional potential reconstruction, virus–host interactions, pathway selection, strain dispersal, and novel genome discoveries. However, there still remain pitfalls and limitations from conventional metagenomic analysis being applied to halophilic microbial communities. Deconvolution of halophilic metagenomes has been difficult due to the high G + C content of these microbiomes and their high intraspecific diversity, which has made both metagenomic assembly and binning a challenge. Halophiles are also underrepresented in public genome databases, which in turn slows progress. With this in mind, this review proposes experimental and analytical strategies to overcome the challenges specific to the halophilic microbiome, from experimental designs to data acquisition and the computational analysis of metagenomic sequences. Finally, we speculate about the potential applications of other next-generation sequencing technologies in halophilic communities. RNA sequencing, long-read technologies, and chromosome conformation assays, not initially intended for microbiomes, are becoming available in the study of microbial communities. Together with recent analytical advancements, these new methods and technologies have the potential to rapidly advance the field of halophile research.

## 1. Introduction 

**Figure 1:**
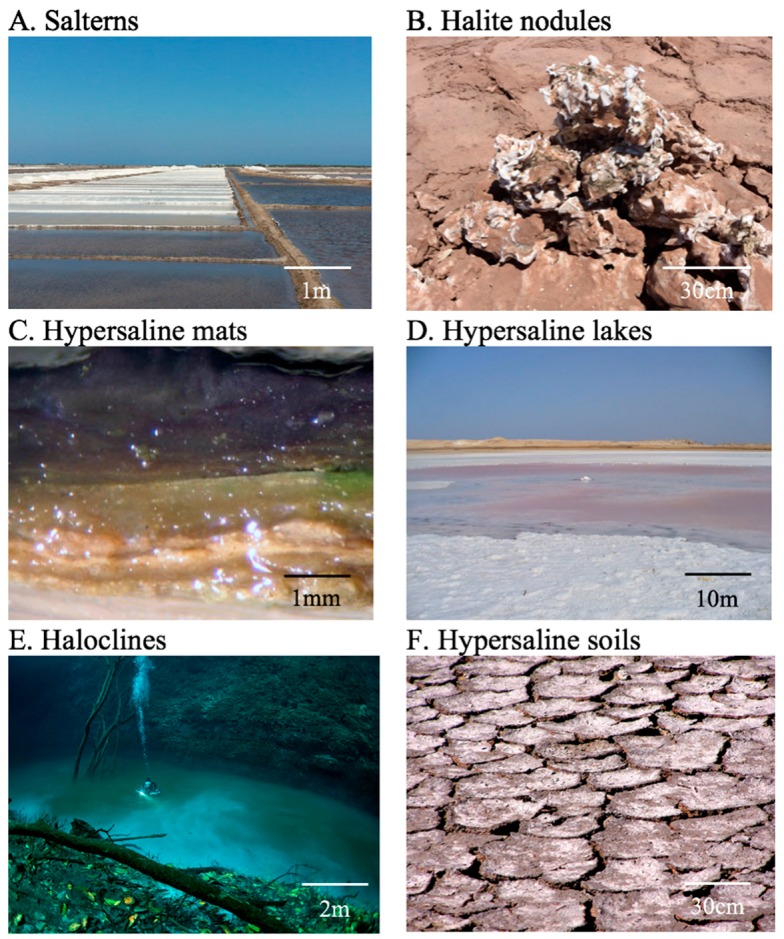
Photographs of commonly studied hypersaline environments: (**A**) saltern flats, (**B**) halite nodules, (**C**) hypersaline microbial mats, (**D**) hypersaline lakes, (**E**) underwater haloclines, and (**F**) hypersaline soils. * Sources for images (free-to-use sources): https://commons.wikimedia.org/wiki/File:Salterns,_salt_making_fields,_tamil_nadu_-_panoramio.jpg, https://en.wikipedia.org/wiki/Phototrophic_biofilm#/media/File:Microbial_mat_section.jpg, https://commons.wikimedia.org/wiki/File:Saline_Lake_at_Ras_Mohamed_National_Park.jpg, https://commons.wikimedia.org/wiki/File:Halocline.png, https://pxhere.com/en/photo/1132612.

Microbial life is one of the most diverse and bioenergetically dominant forces in the earth’s ecosphere [1], making microbiome research a critical component of modern ecology. The unparalleled taxonomic and functional diversity of microbial communities has allowed them to populate all locations on the planet [2,3], including environments unfit for colonization by other life forms. In hypersaline environments, unique environmental pressures have forced microbiota to evolve with specific survival adaptations, resulting in highly resilient communities that push the boundaries of life’s limit (Figure 1). Halophiles have been found to play important roles in soil bioenergetic processes [4] and food storage and preservation [5,6], and have also been detected in the human gut microbiota [7]. Additionally, studying halophilic life forms has revealed many fundamental aspects of life’s survival limits and strategies, including the potential to endure the harsh environments we are most likely to find on other planets [8,9]. Prior to the introduction of high-throughput sequencing, our understanding of halophile genomics was limited to studying cultured organisms [10,11]. While next-generation sequencing technologies have become commonplace in microbiology, the halophile field lacks a critical analysis of prospects and potential applications of these technologies in halophilic microbiomes. 

In this review, we discuss key aspects of halophile community composition and function that metagenomics has revealed and provide examples of studies in various hypersaline environments for a perspective on analytical progress. We then examine the advantages and limitations of applying shotgun metagenomic sequencing in uncovering the structure and function of halophilic microbiomes. We outline the factors and characteristics that make the deconvolution of halophilic metagenomes a major challenge and propose analytical adjustments to be made when investigating these complex communities. Both experimental design and computation analysis approaches that are appropriate in halophilic metagenomics are summarized. Finally, we discuss novel sequencing technologies that show promise in further propelling the halophile metagenomic field. 

## 2. Shotgun Sequencing in Metagenomics

Rapid developments in high-throughput DNA sequencing technologies since the early 2000s have propelled our understanding of not only single-organism genetics, but also microbiome community structure and function [12]. Marker gene (particularly the *16S rRNA* gene) amplicon sequencing has revealed the taxonomic composition of a given community through sequencing a small target of the community’s DNA. In contrast, whole-metagenomic sequencing (WMGS) theoretically allows for reconstruction of the entire microbial community’s DNA content. This has led to a number of important findings in microbiome research [12,13,14], as biologists have been able to thoroughly investigate microbial communities at the genetic level without the need for culturing [15]. 

However, while sequencing technologies are rapidly developing, producing complete genomes of all the microorganisms found in a community is currently unattainable due to low sequencing coverage of the less abundant organisms. Additionally, sequence repeats and regions of homology between organisms limits genome recovery from short-read data, resulting in incomplete assemblies. Instead, long contiguous pieces (contigs) of genomes are produced, ranging in length from 1 Kbp to 1 Mbp [16,17]. These contigs then need to be grouped based on the genome they belong to, a process known as binning. It is only recently that binning has become reliable enough to produce reasonably high-quality metagenome-assembled genomes (MAGs). The ability to produce high-quality MAGs has in turn led to the discovery of thousands of novel organisms and has thus enabled many breakthroughs in characterizing the taxonomic and functional components of microbiomes [18,19,20].

Shotgun metagenomics offers tremendous advantages in recovering taxonomic and functional potential components of microbial communities, but sequencing costs deter some researchers from deploying this approach in their studies. The high average read coverage required for the assembly of a genome from shotgun reads [21] presents a major challenge for the assembly of less-abundant organisms in a metagenomic context. These highly diverse but underrepresented taxa often constitute significant proportions of microbial communities and play important roles in biome functioning [22]. Despite these challenges, WMGS carries tremendous benefits, empowering researchers to study previously unknown aspects of microbiomes. In particular, WMGS allows for the reconstruction of a given community’s gene content, which has enabled ecologists to predict the functional potential of entire communities. This new angle of microbiome analysis has enabled the prediction of metabolic processes potentially present in communities and the study of community natural selection at the functional level [23,24]. The possibility of studying the functional potential of any organism in a community means that our understanding of microbial genetics, dynamics, evolution, and function is no longer limited to cultured organisms. In many fields, such as human microbiome research, this has hailed a new era for research [25,26]. 

### 2.1. Halophilic Microbiome Research Powered by Shotgun Metagenomics

Numerous breakthroughs in halophilic microbiome research have been enabled by WMGS [11] (Table 1). This sequencing approach reveals the taxonomic structure of microbiomes in high-salt environments with significantly less taxonomy-based biases than conventional ribosomal amplicon sequencing. Indeed, in conventional *16S rDNA* amplicon sequencing, primer choices can have a substantial impact on taxonomic distribution, and it is difficult to reliably amplify multiple domains of life, e.g., Bacteria and Archaea, with the same primer set [27]. While WMGS still has biases associated with G + C content, taxonomic annotation of shotgun reads usually results in more accurate and robust taxonomic profiles than amplicon sequencing [28]. This is particularly important in high-salt environments, where both Archaea and Bacteria are found in high abundance. For example, shotgun sequencing has provided more comprehensive taxonomic profiles of an endolithic halite community (Figure 1B) and the discovery that a unique algae was present in this community, in addition to Halobacteria, Cyanobacteria, and other heterotrophic bacteria [29]. In the study of a hypersaline lake (Figure 1D), the use of shotgun sequencing revealed the functional redundancy between taxonomically dissimilar communities constituted of both bacteria and archaea along a salinity gradient [30]. WMGS also provides DNA sequences that are not targeted by *16S rDNA* amplification, including eukaryotic genomes, DNA viruses, and extrachromosomal DNA, such as plasmids. For example, in a study investigating the community composition of saltern ponds (Figure 1A) along a salinity gradient, the use of metagenomics allowed access to both the cellular and viral components of the community within the same sequencing datasets, revealing increased virus abundance at higher salt concentrations [31].

The reconstruction of viral genomes from hypersaline environments [32] using WMGS has resulted in improved characterization of this major component of halophilic microbiomes. Viruses take on the vital role of predators in many microbiomes and contribute to nutrient turnover with their lytic activity [33,34]. While nonshotgun approaches have been used previously to characterize halophilic metaviromes [35,36], high-throughput sequencing has empowered a more streamlined and unbiased recovery and annotation of viral sequences from various types of high-salt environments (Table 1). For example, an investigation of the metavirome in deep-sea haloclines (Figure 1E) through nontargeted shotgun sequencing revealed the stratification of virus lineages along the salinity gradient of the haloclines, likely associated with their host specificity [37]. In WMGS from solar salterns (Figure 1A), perfect alignments between the CRISPR spacers of microorganisms and viral sequences have been used together with di- and trinucleotide frequencies to predict and validate host specificity among halophilic phages across several locations [38]. Another study looking at halophilic *Cyanobacteria* in endolithic communities (Figure 1B) used virus sequences encoded in CRISPR arrays as a high-sensitivity strain signature, which allowed for the tracking of strain dispersal in the region [39].

As previously mentioned, one of the biggest strengths of WMGS is the ability to reconstruct the functional potential of a microbial community. With WMGS, hypersaline water [8,40], soil [4], and endolithic [41] microbiomes have been characterized in terms of their metabolic function, particularly their ability to use a wide range of energy sources. In particular, building on previous culture-dependent methods, systematic functional analysis of halophilic metagenomes has led to major improvements in our understanding of halophile osmotic adaptation and evolution [42]. For example, longitudinal analysis of halite endolith (Figure 1B) microbiota after a heavy rainfall revealed metaproteome adaptations to the temporarily decreased salt concentrations [41]. Functional annotation of longitudinal studies of halophiles from saltern, hypersaline lake, and salt mineral environments has also led to the characterization of horizontal gene transfers, evolutionary dynamics, and functional adaptations across time and space [40,41,43,44]. Functional potential profiling has also uncovered selective pressures and community functional dynamics that were not possible to investigate through taxonomy alone due to high functional redundancy. For example, the investigation of metagenomes from hypersaline soils (Figure 1F) has allowed researchers to uncover core differences in the functioning of their communities compared to more homogeneous aquatic hypersaline environments, which stems from nutrient scarcity, limited mobility, and niche stratification [4]. In a metagenomic study of phototropic hypersaline microbial mats (Figure 1C), functional annotation and pathway quantitation led to a better understanding of energy and nutrient capture and cycling between layers of the mats [45]. In particular, identification of MAGs with complementary parts of nitrogen and sulfur metabolism pathways suggested a dependence on the metabolite exchange between community members. A functional potential investigation of microbial communities of solar saltern ponds (Figure 1A) revealed a higher prevalence of DNA replication and repair machinery in communities found in saturated brine compared to subsaturated saline environments [31]. With WMGS analysis rapidly improving and halophile databases rapidly growing [46], more breakthroughs will follow. 

Another major aspect of metagenomics facilitated by WMGS is the reconstruction of novel individual genomes of halophiles. This is particularly important because extreme halophiles, and extremophiles in general, have been difficult to isolate due to specific growth condition requirements, symbiotic relationships, and cross-species functional pathways [47]. The binning of metagenomics assemblies has enabled researchers to recover hundreds of halophilic MAGs in the past decade [46], with many belonging to previously unknown orders, or even phyla [48]. For example, metagenomic binning of WMGS data from Lake Tyrel resulted in the recovery of near-complete genomes from a new clade of Nanohaloarchaea [49]. Similarly, metagenomic binning of solar saltern metagenomes uncovered several novel lineages of Euryarchaeota, Nanohaloarchaea, and Gammaproteobacteria. Functional annotation of these novel lineages allowed researchers to infer their metabolic functions within the microbiome [50]. In a halite endolith (Figure 1B) longitudinal study following a rare rain, community composition at the strain level was interrogated by genome-resolved metagenomics, leading to a general model of fine-scale taxonomic rearrangement of microbial communities following acute perturbations [41]. In addition to these individual discoveries, the rapidly increasing number of annotated reference halophile genomes allows for more accurate taxonomic and functional annotation in halophilic microbiomes, propelling the field in a positive-feedback loop [46]. 

### 2.2. Limitations of Shotgun Metagenomics in Halophile Research

In contrast to human and synthetic microbiomes, the reconstruction of environmental metagenomes has been complicated by their sheer diversity and microdiversity. This is especially true in high-salt environments, which often host microbial communities with low taxonomic diversity but very high intraspecific diversity and characteristically high G + C content [68,69]. The presence of a large number of highly similar strains presents major challenges for deconvoluting their DNA content during metagenomic assembly and binning. This is particularly problematic in many halophiles that have genomic island regions of high inter-strain variability stemming from horizontal gene transfer [70,71]. On the other hand, the high G + C content of many dominant halophiles reduces the fraction of unique sequences in the samples [56,72], posing another challenge at the assembly stage. For example, halophilic endolith communities are typically dominated by Halobacteria and Salinibacter, but their high strain diversity and G + C content (over 60%) leads to relatively poor assembly and MAG quality [32]. In contrast, other community members that are less abundant and have low G + C content, such as Cyanobacteria, Actinobacteria, and Gammaproteobacteria, have yielded high-quality MAGs [41]. 

Due to the previously mentioned difficulties in culturing a diversity of halophiles, there are a relatively small number of genomes available. In 2018, there were just 942 complete halophile genomes available in NCBI databases [46], a tiny number in the era of high-throughput sequencing, which thus far has yielded over 200,000 prokaryotic complete genomes [73]. This leaves MAG extraction from environmental sequencing data the primary method for obtaining genomes of halophilic organisms, which has been difficult because of their metagenomic properties. In a negative feedback loop, this in turn has further stalled the progress of halophilic microbiome research, as the lack of available reference genomes has made taxonomic and functional annotation difficult. As WMGS becomes commonplace in microbiome research, it is crucial that the halophile field takes full advantage of the new technology and the use of newly available bioinformatic tools to further its understanding of microbial community assembly and function. Since 2014–2015, improvements in analytical methods and assembly software such as metaSPAdes [74], binning software such as metaBAT2 [75], and processing pipelines such as metaWRAP [18] have allowed for effective deconvolution of WMGS data from even the most complex microbiomes. These new analytical methods will greatly benefit the halophile research field, if applied effectively.

## 3. Experimental Design Considerations for Sequencing Halophilic Metagenomes

Obtaining MAG-level resolution in a metagenome enables more accurate and meaningful functional pathway and taxonomic annotation and allows for detailed analysis of specific members of the community. With this in mind, the end goal of many microbiome studies is accurate and complete binning of sequence data. There are two general approaches to metagenomic sequencing and analysis for this purpose: (1) co-assembly of multiple shallowly sequenced samples or (2) individual processing of a few deeply sequenced samples. Both approaches have their benefits and limitations, depending on the microbiome that is sequenced and the biological question to answer. 

**Figure 2:**
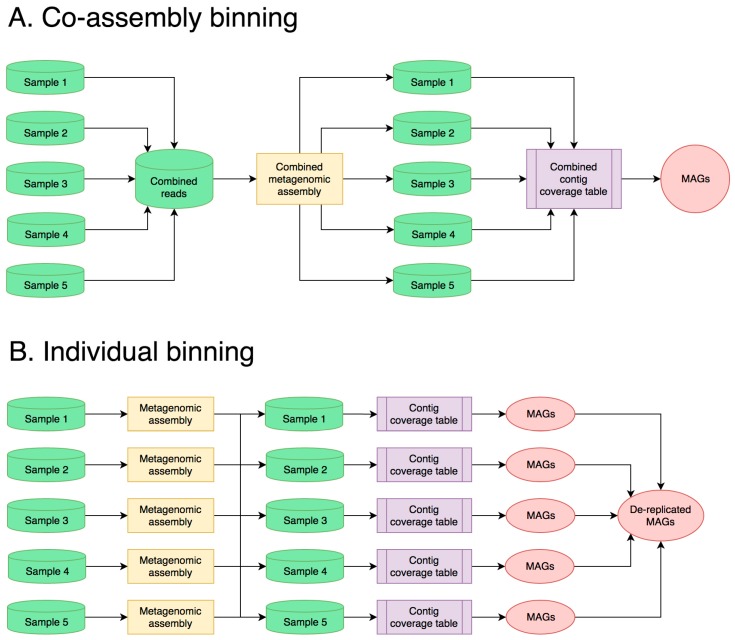
Flowcharts showing two common experimental designs and analysis workflows: (**A**) co-assembly and (**B**) individual sample processing and binning.

In the first approach, samples are sequenced with relatively low-read coverage, and reads from all samples are combined during metagenomic assembly (Figure 2A). In research projects that demand a large number of samples, such as longitudinal studies, this results in low sequencing costs per sample, while also producing high-quality MAGs from the co-assembly by leveraging differential abundances of the contigs across samples [18,75]. The taxonomic and functional composition of individual samples can be investigated by linking the taxonomic and functional annotations of each contig with its abundance in each sample, allowing for easy comparisons between large numbers of samples [41,43]. Finally, co-assembling data from multiple samples enhances the recovery of genomes from low-abundance organisms, which is not possible from individual samples due to low coverage [49]. However, the use of co-assembly in metagenomics comes with significant drawbacks [56], including the high computational costs of co-assembling large data and the high level of microdiversity introduced by each new biological replicate. This latter point might be counterintuitive, but it leads to poor assemblies of very abundant taxa because accumulated mismatches from strain heterogeneity complicate the De Bruijn graph during assembly. This is particularly problematic with halophilic microbiomes, which are often dominated by highly diverse groups of Euryarchaeota and Bacteroidetes [48]. The high population microdiversity of these taxa is exacerbated when using multiple biological replicates, which results in poor, fragmented, or chimeric assemblies [56]. This in turn translates into poor-quality MAGs. However, when a broad capture of community diversity across many samples is the intent of the study, these limitations should then be considered in data interpretation. 

An alternative approach to co-assembly is to sequence a small number of samples with deep coverage and process them individually (Figure 2B). Because of the reduced microdiversity, individual assemblies produce larger contigs, given a comparable sequencing depth [76]. After binning each sample separately, MAGs can be combined into a single set through dereplication, removing duplicate MAGs that share a high nucleotide identity [77]. As with the co-assembly approach, differential contig coverage across samples may be used to improve binning results [40]. While this method is superior in highly heterogeneous communities such as halophilic microbiomes, it comes with a major increase in sequencing cost per sample. For most metagenomes, a meaningful assembly (N50 > 5 Kbp) requires 25–50 Gbp of sequencing data per sample, which limits the number of samples that can be multiplexed on a sequencing run. In turn, the limited replication reduces the effectiveness of binning, which leverages differential coverage of contigs across many samples to increase binning accuracy [78]. For many studies that require a large number of replicates, such as longitudinal studies, the cost of this approach may become prohibitively expensive. 

An additional consideration in choosing a strategy for metagenomic sequencing and analysis is that of intersample community diversity. Communities in aquatic biomes, such as hypersaline lakes or brine ponds, are often more homogenous, harboring the same microorganisms with different relative abundances at different sampling locations. Under those conditions, a co-assembly strategy for metagenomics, as discussed above, is often preferred [43,49,79]. In contrast, in terrestrial microbiomes with limited dispersal, such as halite nodules in salars of the Atacama Desert, which contain unique taxonomic compositions, an individual assembly approach is more advantageous [29,39]. Hybrid approaches are also possible in many cases, as binning of the individual and grouped assemblies may be combined and dereplicated to obtain the most robust MAGs of both rare and abundant species [80]. Regardless of the experimental design, it is critical to process samples, generate libraries, and sequence samples together to avoid batch effects [81]. If more than one flow cell is required to achieve the desired read depth, it is usually better to sequence the pooled libraries on several flow cells than to sequence each sample on its own flow cell [81]. For library preparation, it is recommended to use protocols that produce minimal G + C biases in coverage, particularly in halophilic communities that have high G + C content variation in their metagenomes [82,83].

The take-home message is that, when conducting a halophile metagenomic study, it is especially important to design a sampling and sequencing scheme with statistical questions in mind. Because of the high strain-level diversity typically found in halophilic microbiomes, an experimental design should avoid adding unnecessary replicates into the study, as each added biological replicate will introduce more microdiversity into the data, further complicating the assembly and binning stages of the analysis [56]. In practical terms, unless the intent of the study is to capture maximum diversity, the experimental design should include the minimum number of biological replicates that will allow for the intended statistical analysis downstream. 

### 3.1. Best Bioinformatics Practices for Halophilic Metagenome Analysis

When processing halophilic metagenome sequencing data, it is important to adjust existing pipelines to accommodate for high intraspecific diversity, G + C content diversity, and underrepresentation in most sequence databases. While this section does not provide step-by-step instructions for bioinformatics analysis, it outlines core considerations and adjustments that should be made when processing halophilic metagenomes. Automated metagenomic analysis pipelines such as metaWRAP [18] or SqueezeM [84] may be used to streamline and simplify analysis: However, pipelines that are specifically designed for animal microbiomes, such as gut microbiota, should be avoided. Indeed, these latter pipelines rely strongly on pre-existing taxonomic and functional databases of closely related organisms, as the majority of organisms found in host-associated microbiomes have been sequenced and characterized. 

The preprocessing of WMGS data, which typically includes read trimming, duplicate read removal, and metagenomic assembly, is standard for most types of metagenomes. We encourage testing a variety of software and comparing the results to evaluation programs such as FastQC [85] (for read quality) and MetaQUAST [86] (for assembly quality), as some methods may be more suited to specific microbial community types [87]. For metagenomic assembly, metaSPAdes [74] is currently considered to be the best overall, while MegaHIT [88] is a better solution when resources are a limiting factor, as it is significantly faster and requires less memory [89]. Thanks to recent improvements in assembly software, it is no longer necessary to subsample reads during this stage, as contig quality no longer drops off with increased read depth [89]. However, higher-quality assemblies of abundant organisms can be achieved through individual or grouped sample assembly, as described above.

In contrast to assembly, the annotation of halophilic metagenomes for taxonomies and functions can be somewhat compromised because halophiles have extremely limited representation in standard-distribution taxonomic databases [90,91], which introduces significant biases in sequence annotation. As of 2018, there were only 942 published complete halophilic genomes available in NCBI [46], the main database used as a reference in most taxonomic and functional annotation software. Regarding methods for taxonomic profiling, general alignment-based methods such as MegaBLAST [92] are usually too specific for annotating non-assembled halophilic DNA sequences because they rely on high sequence similarity and skew the annotation toward taxa that are better-represented in the database. To produce more balanced taxonomic annotations given the limited databases, it is recommended to assign taxonomies to assembled contigs based on the genes that they carry and then infer the taxonomy of reads based on their alignment with the contigs. If the intent is to obtain the most accurate taxonomic distribution profile of the community, extracting and annotating marker genes (such as *16S rRNA* genes) with EMIRGE is usually the best alternative [93], as rRNA gene databases are more established and encompass greater taxonomic diversity [94]. 

Functional annotation (the functional categorization of genes) in halophile metagenomes is also severely limited by existing databases, especially compared to human microbiomes. Because many halophilic genes are not annotated in NCBI databases, metagenome-inclusive custom or specific databases are preferred because they contain a greater variety of noncultured organisms. In particular, services such as the “Integrated Microbial Genomes and Microbiomes” systems from JGI [95] include taxonomic and functional annotation models that are trained on user-submitted metagenomic data, including high-quality MAGs. The annotation sensitivity resulting from using the newest metagenomic data is extremely valuable for both functional and taxonomic annotation in relatively understudied systems, such as halophilic microbiomes. Regardless of the database being used, it is important to regularly update to the most recent release, as new organisms are constantly being sequenced. Annotation pipelines geared toward human microbiomes such as HUMANN2 [96] should be avoided, as they rely on the presence of closely related organisms in databases. 

For many metagenomic studies, an important objective is the genome-resolved description of the microbiome of interest, since the analysis of individual MAGs opens up many avenues for more accurate and meaningful functional pathway annotations and strain-level comparative metagenomics. To that extent, the success of metagenomic binning of assemblies depends greatly on software choice, as binning programs perform differently with various data types [18]. Additionally, many popular binning software programs, such as metaBAT1, are trained on gut microbiome data [75], potentially limiting their efficacy in complex halophilic communities. Furthermore, benchmarking of such algorithms is often done on real or synthetic gut microbial communities [87]. Because of this, it is recommended to bin the metagenomic assembly with a variety of the most recent binning software, such as metaBAT2 [75] and CONCOCT [97], and to use a binning consolidation tool, such as metaWRAP or DAS_Tool, to produce the best final bin set [18,98]. When estimating the read coverage of the contigs in a given sample to be fed into the binning algorithms, it is important to remember that they represent collapsed averages of a number of strains. Given the high intraspecific diversity of halophilic microbiomes [56], more accurate abundance estimations could potentially be obtained with slightly relaxed read alignment parameters, allowing for more approximate matches. 

Considering the overwhelming number of metagenomic bioinformatics tools coming out each year, it is difficult to keep up to date with the best analytical methods. In general, we advise testing and benchmarking multiple software programs for each analytical step to determine the best option, as many conventionally used software programs behave unpredictably with halophilic sequence data. For annotation, emphasis should be placed on high sensitivity rather than high precision, given the database limitations. 

### 3.2. The Future of Halophilic Metagenomics

Beyond shotgun sequencing of a microbiome’s DNA content, there exist a number of other sequencing technologies that have become available and may further our understanding of halophilic ecosystems. Studies applying these technologies to more developed microbial fields, such as human gut microbiomes, have shown their great promise and their potential applications in halophilic microbial communities in the near future. 

Conventional Illumina sequencing is limited to short DNA fragments (50 bp–250 bp), as errors accumulate rapidly at higher read lengths. However, read length, together with sequencing coverage, is undoubtedly a major limiting factor for metagenomics sequence assembly. Longer reads result in more accurate assembly and reduced chimeras, while they improve the contiguity of the assembly by allowing the assembly of repetitive DNA elements [99]. Recent sequencing technologies (minION from Oxford Nanopore and SMRT from PacBio sequencing) produce longer DNA fragments compared to Illumina. PacBio is able to consistently produce long reads (N50 up to 10 Kbp) with a relatively high degree of accuracy [100,101], while Nanopore sequencing produces even longer reads (N50 up to 100 Kbp), but with some sacrifices in accuracy [102,103]. Read lengths from these technologies enable not only the sequencing of complete ribosomal genes for improved taxonomic annotation, but also a significant improvement in the accuracy of metagenomics assembly and binning [101,104]. In highly diverse halophilic communities, long reads can help assemble ambiguous regions resulting from taxonomic heterogeneity, drastically improving the quality of the metagenome assembly [104]. Pseudo-single-cell technology from 10X Genomics, which tags each read with a barcode unique to the cell it came from, also shows great promise in halophilic microbiome deconvolution, as it is able to produce strain-specific synthetic long reads originating from single cells [105]. With reported maximum read lengths of over 1 Mbp from Nanopore, long-read technology is rapidly approaching the point where sequencing complete genomes in a single read is theoretically possible [106]. When this becomes reality, it will propel the field of metagenomics into a new post-assembly era. However, the recovery of less abundant taxa will remain a concern given the relatively low throughput of these methods.

Chromosome conformation capture with Hi-C is another technology that shows great promise in the field of halophilic metagenomics. A Hi-C assay crosslinks DNA based on spatial proximity: The chimeric segments resulting from the crosslink events are then sequenced, revealing sections of DNA that are proximal to each other. Conventionally used to indirectly measure the proximity between sections of a genome, Hi-C was successfully applied in 2017 to microbiomes to improve binning predictions [107]. Considering the difficulty of binning halophilic metagenomes due to their heterogeneity, Hi-C could significantly improve halophile MAG extraction. Hi-C-based binning also enables the recovery of extrachromosomal elements such as viral and plasmid DNA, which so far has been difficult to accomplish [108]. Hi-C can also be used to produce DNA proximity maps in individual MAGs for the study of chromatin conformation in prokaryotes at the metagenomic and single-cell scale [108].

Finally, genome-resolved metatranscriptomics (the analysis of a microbial community’s RNA content) has been widely used in a variety of microbiomes to interrogate microbial transcriptional activities [25,109]. Metatranscriptomics has been used in halophile research to characterize carbon cycling in saline soils [110] and has been extensively used to characterize activity in other soil microbiomes [111,112]. However, it remains a largely underdeployed tool in many other high-salt systems, partly due to the difficulty in depleting ribosomal sequences in archaeal RNA. Another major deterrent has been the difficulty in standardizing transcript expression to the abundance of each individual organism in a sample. In other words, if a transcript is more abundant in a given sample, it can be difficult to determine if the organism carrying it is more abundant in that sample, or if it is truly highly expressed. However, with rapid improvements in genome-resolved metagenomic analysis of halophile communities, it is possible that the metatranscriptomic problem can be simplified down to more conventional transcriptome analysis by investigating the transcriptomes of individual MAGs. 

## 4. Conclusion

Successful applications of whole metagenomics in halophilic communities has already led to numerous breakthroughs in our understanding of their functional composition, virus–host interactions, and strain diversity and dispersal, and has allowed for the genome extraction of previously unknown halophiles. However, the genomic qualities and composition characteristics of halophilic communities have made them difficult to deconvolute in a metagenomic context, limiting the information that can be extracted from halophilic shotgun metagenomes. Combined with relatively low numbers of cultures of halophiles, this has led to their underrepresentation in existing taxonomical and functional databases, which has further complicated analysis. While in silico deconvolution of halophilic metagenomes is a challenge, it can be accomplished with analysis workflows that account for the specific characteristics of halophile communities. With proper tuning, rapidly advancing sequencing technology has the potential to reconstruct the complete nucleic acid content of halophilic communities, allowing the halophile field to focus on microbial functional activity and interactions. 

## Figures and Tables

**Table 1:** Studies that have contributed novel aspects of halophilic microbial communities through whole-metagenomic sequencing (WMGS) in hypersaline environments (list is not exhaustive). MAG: metagenome-assembled genome.

Environment	Longitudinal Dynamics	MAG Discovery	Functional Potential	Virus Analysis
Hypersaline lakes	Andrade [51], Tschitschko [44], Podell [52]	Narasingarao [49]	Vavourakis [53], Naghoni [30]	Emerson [54], Tschitschko [44], Ramos-Barbero [55]
Salterns	Plominsky [2]	Ramos-Barbero [56], Ghai [50]	Plominsky [31], Ghai [50]	Moller [38], Di Meglio [57]
Hypersaline microbial mats	Mobberley [45], Berlanga [58]	Mobberley [45]	Mobberley [45], Ruvindy [59], Wong [60]	White [61]
Haloclines	N/A	Speth [62]	Guan [63], Pachiadaki [64]	Antunes [37]
Halite endoliths	Uritskiy [41], Finstad [39]	Finstad [39], Uritskiy [41],	Crits-Christoph [65], Uritskiy [41]	Crits-Christoph [65]
Hypersaline soils	Narayan [66]	Vera-Gargallo [4]	Vera-Gargallo [4], Pandit [67]	NA
